# Experimental Study on Combined Microwave–Magnetic Separation–Flotation Coal Desulfurization

**DOI:** 10.3390/molecules29163729

**Published:** 2024-08-06

**Authors:** Guangming Wang, Zhijun Ma, Zhijing Zhou, Yunsheng Zheng, Liang Cheng

**Affiliations:** 1 College of Mining, Liaoning Technical University, Fuxin 12300, China; guangming19971209@163.com (G.W.); zzj198_1819@163.com (Z.Z.); zys_lgd@126.com (Y.Z.); chengliang9506@126.com (L.C.); 2 College of Materials Science & Engineering, Liaoning Technical University, Fuxin 12300, China

**Keywords:** desulfurization rate, deashing rate, microwave irradiation, magnetic separation, flotation, coal

## Abstract

In order to reduce the content of sulfur and ash in coal, improve the desulfurization and deashing rates, a combined experiment method of microwave magnetic separation-flotation was proposed for raw coal. The desulfurization and deashing rates of three experiment methods, namely, single magnetic separation, microwave magnetic separation, and microwave magnetic separation–flotation, were compared. Taking the microwave magnetic separation–flotation experiment method as the main line, the effects of the microwave irradiation time, microwave power, grinding time, magnetic field intensity, plate seam width, foaming agent dosage, collector dosage, and inhibitor dosage on desulfurization and deashing were discussed, and the mechanism of microwave irradiation on magnetic separation and flotation was revealed. The results show that under the conditions of a microwave irradiation time of 60 s, a microwave power of 80% of the rated power (800 W), a grinding time of 8 min, a plate seam width (the plate seam width of a magnetic separator sorting box) of 1 mm, a magnetic field intensity of 2.32 T, a foaming agent dosage of 90 g/t, a collector dosage of 2125 g/t, and an inhibitor dosage of 1500 g/t, the desulfurization and deashing effect is the best. The desulphurization rate is 76.51%, the sulfur removal rate of pyrite is 96.50%, and the deashing rate is 61.91%. Microwaves have the characteristic of selective heating, and the thermal conductivity of organic matter in coal is greater than that of mineral. Microwave irradiation can improve the reactivity of pyrite in coal, pyrolyze pyrite into high-magnetic pyrite, improve the magnetic properties, and improve the magnetic separation effect. Therefore, microwave irradiation plays a role in promoting magnetic separation. Through microwave irradiation, the positive and negative charges in coal molecules constantly vibrate and create friction under the action of an electric field force, and the thermal action generated by this vibration and friction process affects the structural changes in oxygen-containing functional groups in coal. With the increase in the irradiation time and power, the hydrophilic functional groups of –OH and –COOH decrease and the hydrophilicity decreases. Microwave heating evaporates the water in the pores of coal samples and weakens surface hydration. At the same time, microwave irradiation destroys the structure of coal and impurity minerals, produces cracks at the junction, increases the surface area of coal to a certain extent, enhances the hydrophobicity, and then improves the effect of flotation desulfurization and deashing. Therefore, after the microwave irradiation of raw coal, the magnetic separation effect is enhanced, and the flotation desulfurization effect is also enhanced.

## 1. Introduction

China is a big coal user in the world, and coal is one of the main energy sources in our country. However, the sulfur content in raw coal in some areas is higher than the national standard (St = 1%), and sulfur dioxide and other gases generated during the use of high-sulfur coal will pollute the environment, form acid rain and photochemical smog [1], and cause great harm to the human body. When using coal with too high ash content, it will increase the difficulty of cleaning the combustion equipment but also reduce the combustion efficiency of coal and increase the emission of environmental pollutants, so coal desulfurization and ash reduction is particularly important. The green and environmentally friendly production mode advocated in the context of “double carbon” goals [2,3,4] in the new era has good social and economic benefits. Therefore, it is of great significance to seek an experimental method that can improve the comprehensive utilization rate of coal and reduce ash with high efficiency.

Studies have shown that the main methods for coal desulfurization and ash reduction are physical [5,6,7], chemical [8,9,10], and biological methods [11,12,13]. Physical desulfurization can effectively remove inorganic sulfur from coal, and magnetic separation, flotation, and other methods have been industrialized. Chemical desulfurization can effectively remove inorganic sulfur and part of organic sulfur, but it will destroy the structure of coal and the cost is high. Microbial desulphurization can remove part of inorganic sulfur and organic sulfur, but the cycle is long and the efficiency is low, so the physical desulphurization method is mainly chosen in this experiment. Qingfeng Wang et al. [14] adopted the experiment method of combined magnetic separation–flotation desulfurization and ash reduction. Under the optimum conditions of a grinding time of 4 min, a magnetic field intensity of 1.45T, pulse times of 25 times/min, a coal slurry concentration of 80 g/L, a foaming agent dosage of 90 g/t, a collector dosage of 2125 g/t, and an inhibitor dosage of 1500 g/t, the desulfurization rate is 52.25% and the deashing rate 52.63%. Zhenxing Zhang et al. [15] adopted a combined microwave magnetic separation desulfurization experiment method. Under the optimal condition of microwave irradiation for 4 min and a field intensity of 0.5 T, the desulfurization rate is 52%. Linshun Yang [16] adopted the combined microwave–flotation desulfurization experiment method. Under the optimal conditions of a ball milling time of 20 min, ultrasonic pretreatment for 10 min, a pulp concentration of 50 g/L, a dosage of the foaming agent sec-octanol of 100 g/t, a collector BET (diethyl phthalate) of 200 g/t, an inhibitor CaO of 2000 g/t, and a bubble scraping time of 2 min, the desulfurization rate is less than 30%, and the deashing rate is less than 60%. The existing research shows that physical desulfurization has become the main method of desulfurization and deashing, but the desulfurization and deashing rate is limited.

Aiming at the limitation of a low desulfurization and deashing rate in the physical desulfurization process, the combined process of microwave magnetic separation–flotation was proposed. We explore the effects of the microwave irradiation time, microwave power, grinding time, magnetic field intensity, plate seam width, foaming agent dosage, collector dosage, and inhibitor dosage on desulfurization and deashing effect. The influence mechanism of microwave irradiation on magnetic separation and flotation is also analyzed.

## 2. Results

### 2.1. Wet Strong Magnetic Experiment

#### 2.1.1. Grinding Ore Fineness Experiment

**Figure 1:**
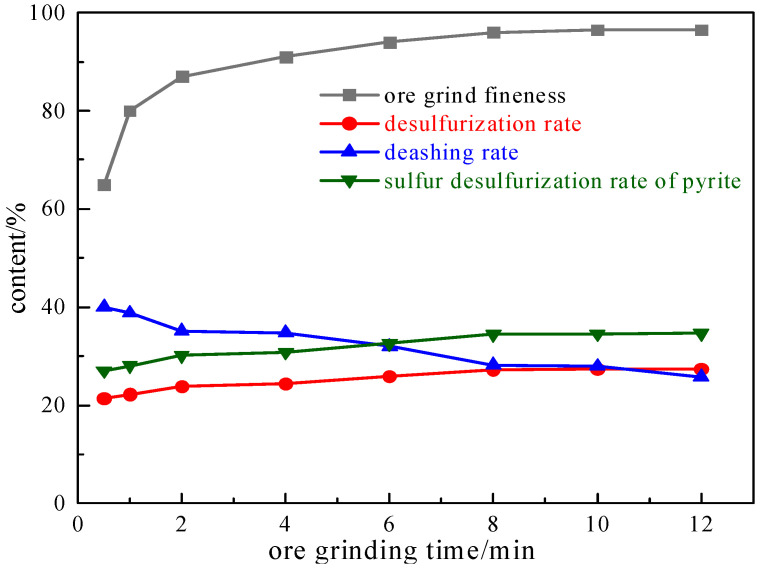
Grinding ore time curve chart.

Taking the coal samples, we investigated the effect of different ore grinding times on grinding fineness under the condition of a magnetic field strength of 2.32 T and a plate seam width of 1 mm. The results are shown in Figure 1.

As can be seen from the curve in Figure 1, with the increase in grinding time, the sample content with a −0.074 mm particle size keeps increasing, and the grinding fineness increases slowly after 8 min. The reason for the improvement in the desulphurization rate of pyrite and the total sulfur desulphurization rate is that with the increase in the grinding time, the pyrite and coal are separated to the maximum extent, which is conducive to the magnetic separation of pyrite and coal, thus improving the desulphurization efficiency. Guided by both grinding fineness and economic cost, the grinding time is selected as 8 min.

#### 2.1.2. Magnetic Separator Plate Seam Width Experiment

**Figure 2:**
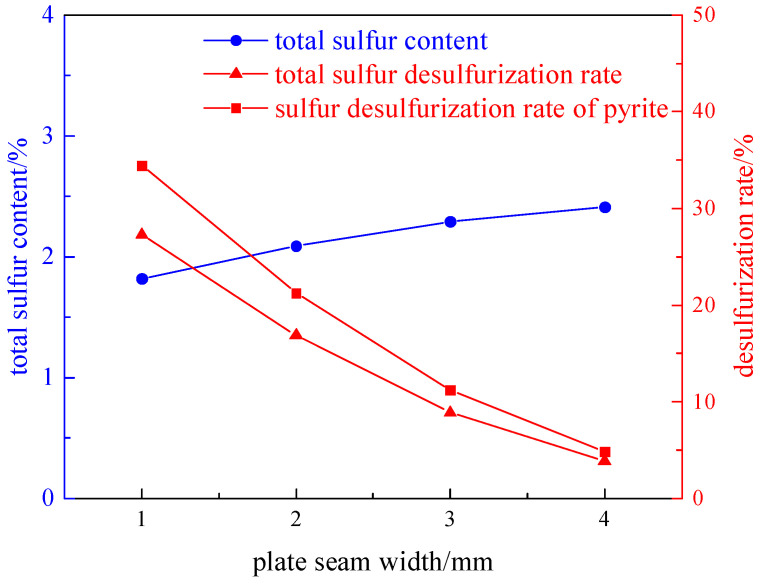
Curve chart of desulfurization effect changing with plate seam width.

**Figure 3:**
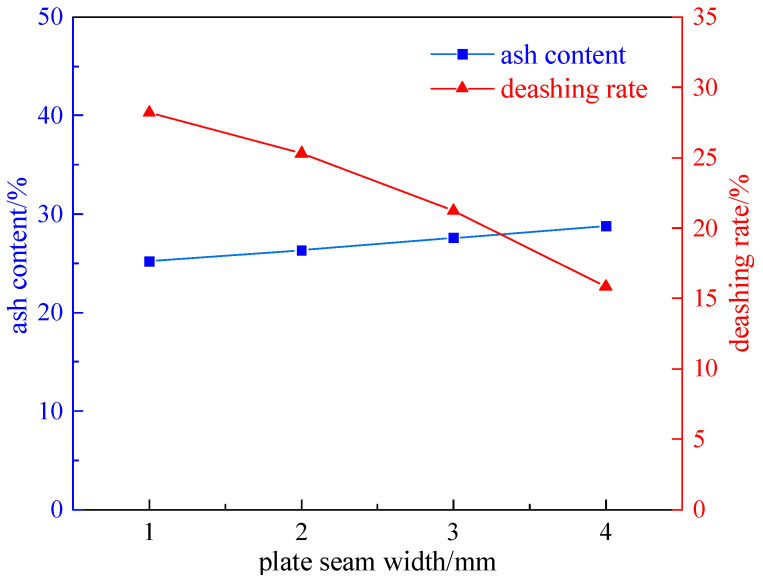
A curve chart of the effect of deashing with the variation in the board seam width.

Taking a 200 g coal sample with a grinding time of 8 min, the effects of different plate seam widths (1 mm, 2 mm, 3 mm, and 4 mm) on desulfurization and deashing were investigated under the condition of a 2.32 T magnetic field intensity. The experimental results are shown in Figure 2 and Figure 3.

From Figure 2 and Figure 3, it can be seen that as the width of the plate seam increases, the total sulfur desulfurization rate and pyrite sulfur desulfurization rate decrease. The reason is that the magnetic field strength is affected by the width of the plate seam, and the larger the width, the smaller the magnetic field strength. Therefore, when the width of the plate seam is 1 mm, the desulfurization rate reaches the highest and the effect is the best; the total sulfur desulfurization rate is 27.30%, the pyrite sulfur desulfurization rate is 34.43%, and the deashing rate is 28.20%.

#### 2.1.3. Magnetic Field Strength Experiment

**Figure 4:**
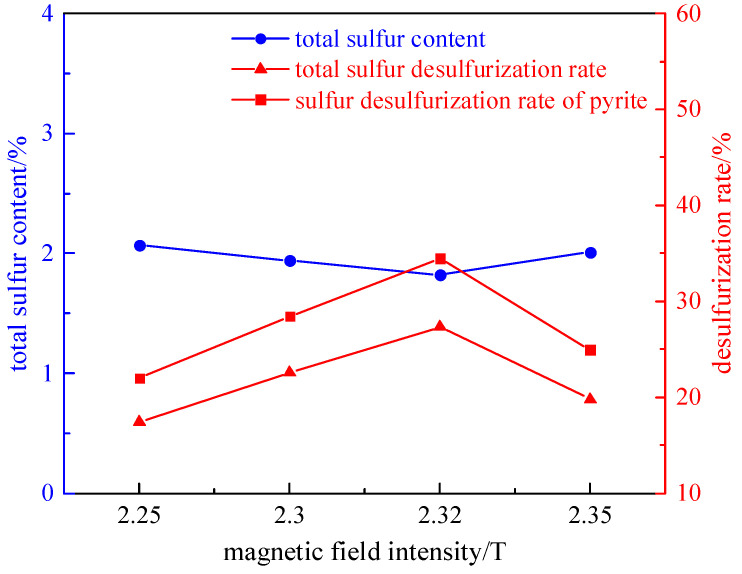
Curve chart of desulfurization effect with field strength variation.

**Figure 5:**
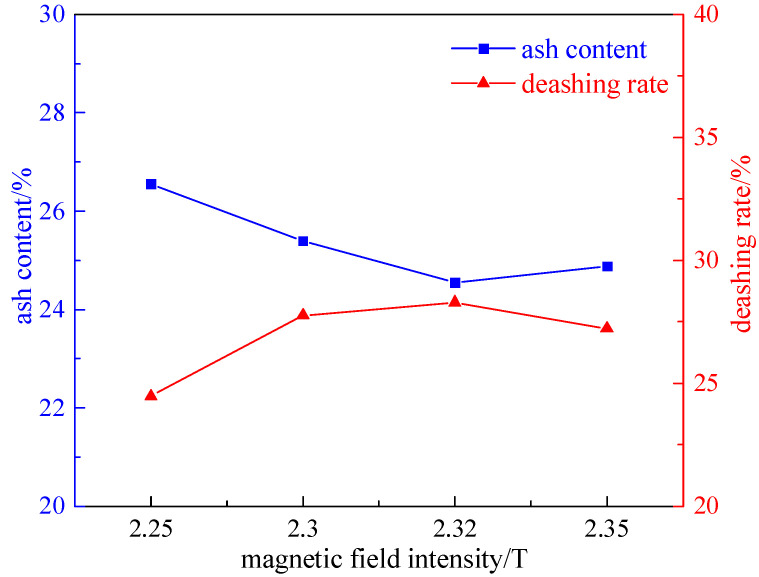
Curve chart of deashing effect with field strength variation.

Taking 200 g of a coal sample with a grinding time of 8 min, we investigated the effect of different magnetic field intensities (2.25 T, 2.3 T, 2.32 T, and 2.35 T) on desulfurization and deashing, under the condition of a plate seam width of 1 mm. Among them, the magnetic separator currents corresponding to the magnetic field intensity are 18 A, 20 A, 22 A, and 24 A successively, and the experimental results are shown in Figure 4 and Figure 5.

As shown in Figure 4 and Figure 5, with the increase in field strength, the desulfurization rate and pyrite sulfur removal rate show a trend of first increasing and then decreasing. The reason is that the separation is based on the difference between the diamagnetism of coal and the paramagnetism of pyrite and the other minerals in coal. With the increasing field strength, the paramagnetic pyrite and inverse magnetic coal are separated, and the magnetic separation efficiency is improved. However, the high-magnetic field will form particle clusters due to magnetic flocculation, and adjacent particles will adhere to each other. Part of the pyrite particles are coated with coal particles, forming almost non-selective flocs that enter the clean coal, resulting in a decrease in desulfurization efficiency. Therefore, the optimal magnetic field intensity of 2.32 T, total sulfur desulfurization rate of 27.33%, pyrite sulfur desulfurization rate of 34.47%, and deashing rate of 28.28% were determined.

### 2.2. Microwave Magnetic Separation Experiment

Traditional heating is based on the thermal conduction of substances, from the surface to the inside, and has no selectivity. Compared with traditional heating, microwaves directly enter the interior of the medium in the form of medium loss wave energy [17,18,19]. The heating effect on the medium varies according to the different tangent values of medium loss, showing selectivity for heating various substances [20,21,22]. At the same time, microwave heating also has a non-thermal effect, which can reduce the activation energy of the reaction and accelerate the reaction. Due to the fact that the direct magnetic separation desulfurization effect does not meet the practical application requirements, microwave irradiation is used to pretreat the raw coal, enhancing the magnetism of pyrite and achieving a good desulfurization effect [23]. Therefore, microwave irradiation conditions are added before magnetic separation, and the effects of the irradiation time and irradiation power are discussed.

#### 2.2.1. Irradiation Time Experiment

**Figure 6:**
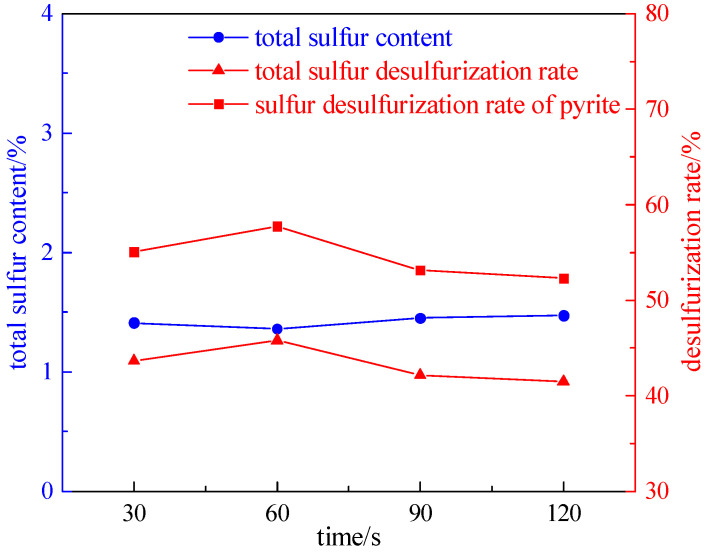
A curve chart of the desulfurization effect changing with the irradiation time.

**Figure 7:**
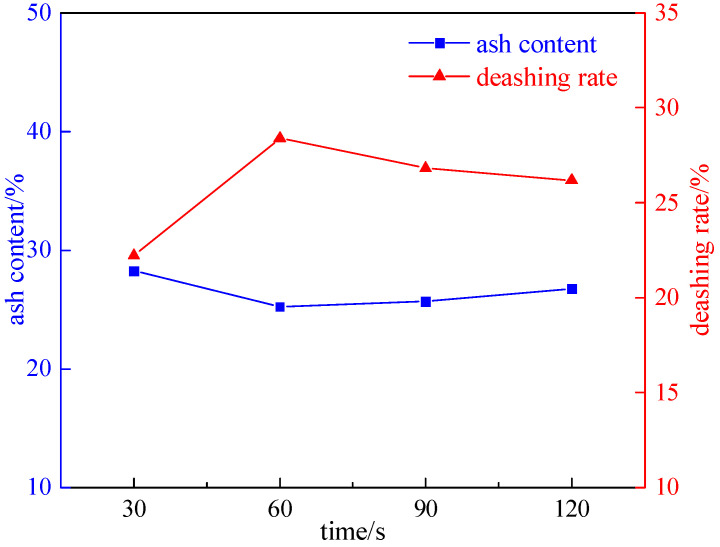
A curve chart of the effect of deashing on the irradiation time.

Taking a coal sample with a grinding time of 8 min, we investigated the effect of the irradiation time (30 s, 60 s, 90 s, and 120 s) on desulfurization and deashing, under the conditions of a magnetic field strength of 2.32 T, plate seam width of 1 mm, and microwave power of 50% of the rated power. The experiment results are shown in Figure 6 and Figure 7.

As shown in Figure 6 and Figure 7, with the increase in the irradiation time, the total sulfur desulfurization rate and pyrite sulfur desulfurization rate first increase and then decrease. The reason is that the pyrite in the coal will be pyrolyzed into magnetite with strong magnetism. If the irradiation time continues to increase, the pyrite will decompose into siderite, which has almost no magnetism, and the magnetic separation effect will deteriorate. Therefore, the optimal irradiation time is 60 s, the total sulfur desulfurization rate is 45.79%, the pyrite sulfur desulfurization rate is 57.75%, and the deashing rate is 28.40%.

#### 2.2.2. Irradiation Power Experiment

**Figure 8:**
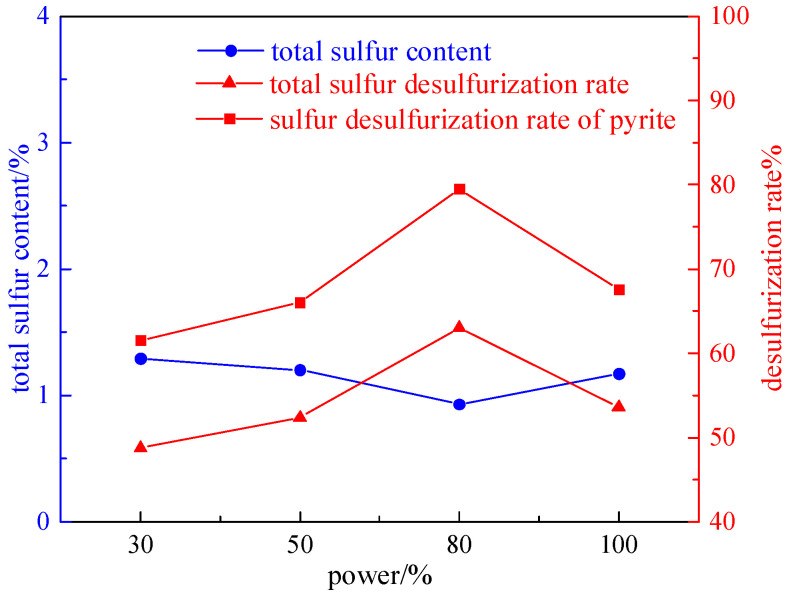
Curve chart of desulfurization effect changing with microwave power.

**Figure 9:**
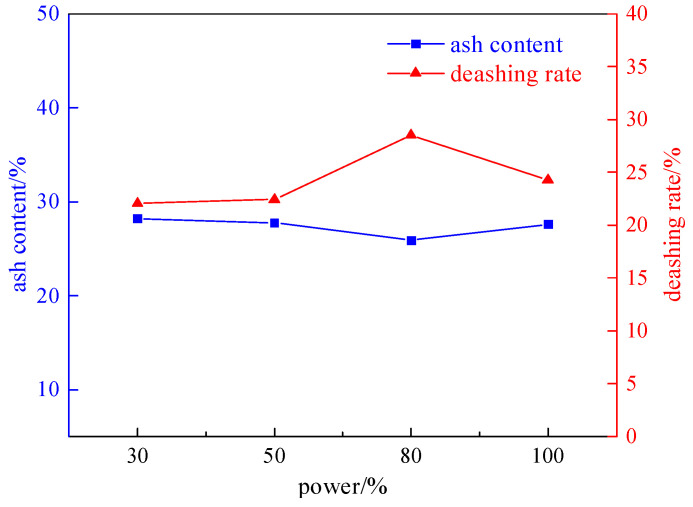
Curve chart of deashing effect changing with microwave power.

Taking coal samples with a grinding time of 8 min, under the conditions of a magnetic field strength of 2.32 T, a plate seam width of 1 mm, an irradiation time of 60 s, and a microwave power of 800 W (30%, 50%, 80%, and 100%), the effect on desulfurization and deashing was studied. The experiment results are shown in Figure 8 and Figure 9.

From Figure 8 and Figure 9, it can be seen that with the increase in microwave power, the total sulfur desulfurization rate and pyrite sulfur removal first increase and then decrease. The reason is that the increase in microwave power is beneficial for improving the reactivity of pyrite in coal, converting it into magnetic minerals, and further increasing the microwave power leads to an increase in siderite and a decrease in magnetic separation efficiency. Therefore, the optimal microwave power is 80% of the rated power, with a total sulfur desulfurization rate of 63.02%, a sulfur removal rate of 79.49% for pyrite, and a deashing rate of 28.51%.

According to Table 1, after microwave irradiation, the microwave magnetic separation test showed a 35.69% increase in the total sulfur desulfurization rate and a 45.02% increase in the pyrite sulfur desulfurization rate compared to the single magnetic separation test, with significant effects. Therefore, the microwave magnetic separation test is more optimal.

### 2.3. Microwave Magnetic Separation–Flotation Experiment

In order to further improve the desulfurization rate and increase the flotation experimental process, a combined microwave magnetic separation–flotation desulfurization and deashing experiment was conducted. The factors affecting the effectiveness of flotation experiments include the dosage of the foaming agent, collector, and inhibitor, with pine alcohol oil as the foaming agent, kerosene as the collector, and calcium oxide as the inhibitor.

#### 2.3.1. Foaming Agent Dosage Experiment

**Figure 10:**
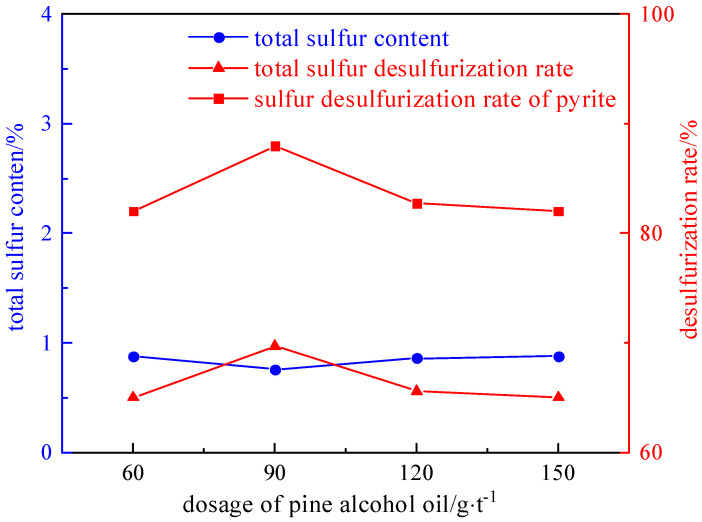
Curve chart of desulfurization effect changing with foaming agent dosage.

**Figure 11:**
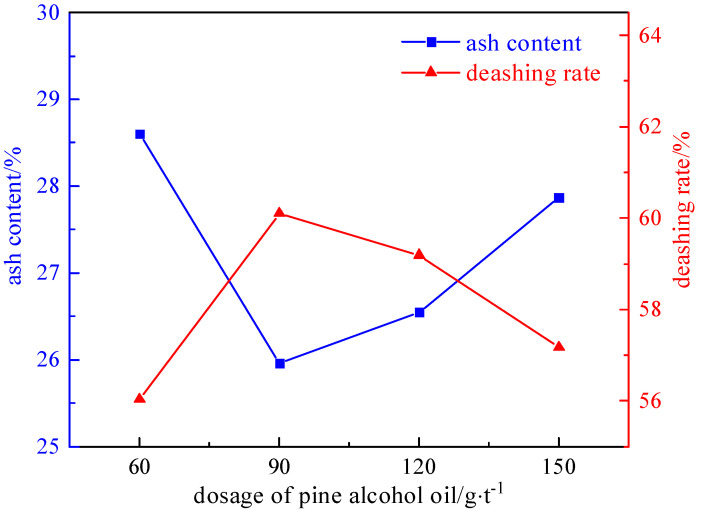
Curve chart of deashing effect changing with foaming agent dosage.

Under the conditions of a slurry concentration of 50 g/L, a collector dosage of 2125 g/t, and an inhibitor dosage of 1000 g/t, the influence of the foaming agent dosage (60 g/t, 90 g/t, 120 g/t, and 150 g/t) on the desulfurization and deashing effect was investigated. The experimental results and relationship curves are shown in Figure 10 and Figure 11.

As shown in Figure 10 and Figure 11, the desulfurization rate shows a trend of first increasing and then decreasing with the increase in the foaming agent. The reason is that the foaming agent promotes the formation of mineralized bubbles during the flotation process, and the clean coal floats out with the bubbles. However, when the amount added is too high, some pyrite also floats out with the bubbles, resulting in a decrease in the desulfurization rate. Therefore, the optimal dosage of the foaming agent is 90 g/t. The desulphurization rate of the total sulfur is 69.72%, the desulphurization rate of the pyrite is 87.94%, and the deashing rate is 60.10%.

#### 2.3.2. Collector Agent Dosage Experiment

**Figure 12:**
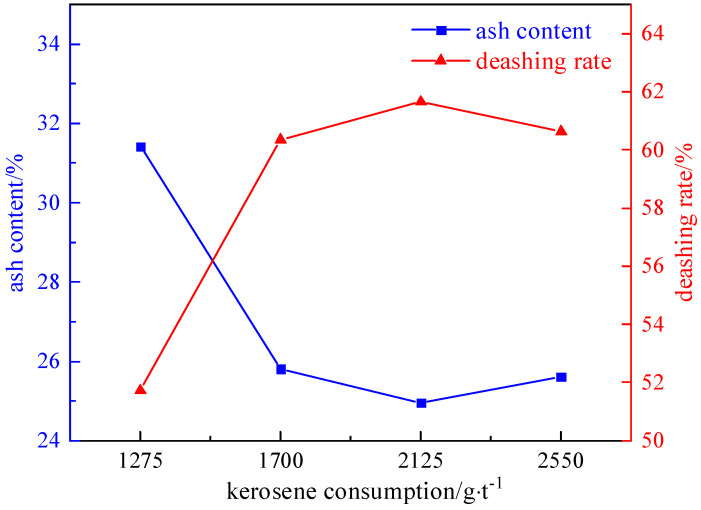
Curve chart of desulfurization effect changing with collector dosage.

**Figure 13:**
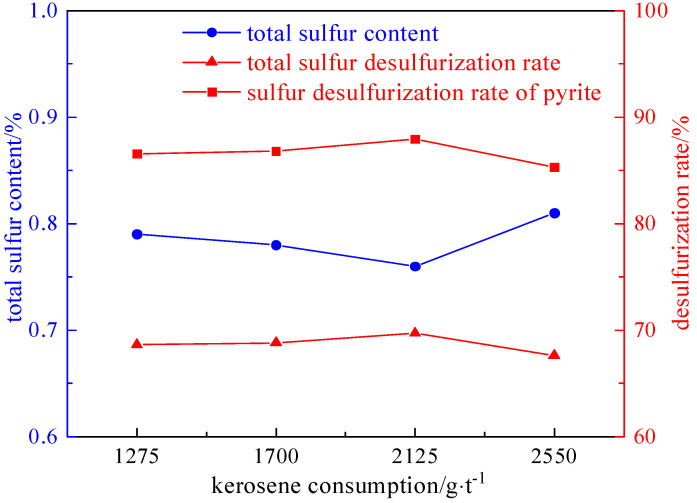
Curve chart of deashing effect changing with collector dosage.

Under the conditions of a slurry concentration of 50 g/L, a foaming agent dosage of 90 g/t, and an inhibitor dosage of 1000 g/t, the influence of the collector dosage (1275 g/t, 1700 g/t, 2125 g/t, and 2550 g/t) on the desulfurization and deashing effect was investigated. The experimental results and relationship curves are shown in Figure 12 and Figure 13.

As can be seen from Figure 12 and Figure 13, the desulphurization rate increases first and then decreases with the increase in the collector. This is because the collector acts at the solid–liquid interface and selectively adsorbs on the surface of minerals to improve the hydrophobicity and floatability of the mineral surface, so that the floating ore particles adhere to the bubbles and enhance the fastness of the two attachments, thus increasing the desulphurization rate. But as the amount of kerosene increases, the system’s ability to capture minerals increases, and pyrite is used as a floating substance to enter the clean coal, resulting in a decrease in the desulfurization rate. Therefore, the optimal amount of collector was determined to be 2125 g/t. The desulfurization rate of the total sulfur is 69.75%, the sulfur removal rate of the pyrite is 87.97%, and the ash removal rate is 61.66%.

#### 2.3.3. Inhibitor Dosage Experiment

**Figure 14:**
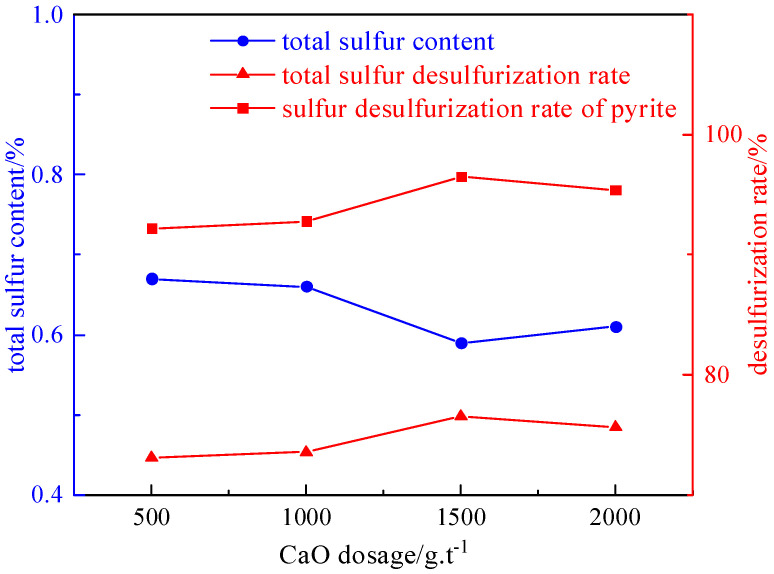
Curve chart of desulfurization effect changing with inhibitor dosage.

**Figure 15:**
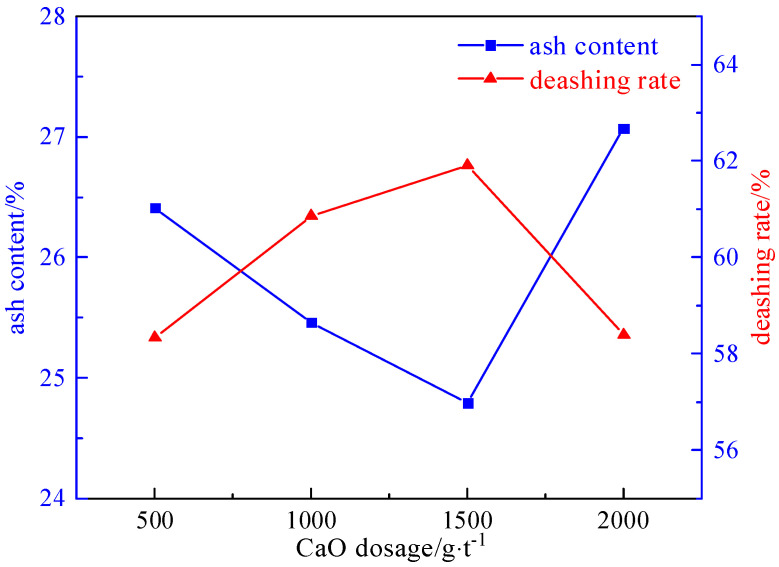
Curve chart of deashing effect changing with inhibitor dosage.

Under the conditions of a slurry concentration of 50 g/L, foaming agent dosage of 90 g/t, and collector dosage of 2125 g/t, the influence of the inhibitor dosage (500 g/t, 1000 g/t, 1500 g/t, and 2000 g/t) on the desulfurization and deashing effect was investigated. The experimental results and relationship curves are shown in Figure 14 and Figure 15.

From Figure 14 and Figure 15, it can be seen that the desulfurization rate shows a trend of first increasing and then decreasing with the increase in inhibitors. The reason is that coal has strong floatability, and it is not easy to mix evenly when preparing slurry. When a small amount of inhibitor is added, calcium oxide selectively acts on the mineral surface, inhibiting the floatability of the sulfide minerals. This results in coal and pyrite being mixed evenly in the water and improves the sorting efficiency. But if the dosage of inhibitors is too high, the floatability of some clean coal particles is inhibited, leading to a decrease in the desulfurization rate. Therefore, the optimal dosage of calcium oxide is determined to be 1500 g/t. The desulphurization rate of the total sulfur is 76.51%, the sulfur removal rate of the pyrite is 96.5%, and the deashing rate is 61.91%.

According to Table 2, the combined microwave magnetic separation–flotation desulfurization experiment has the best effect. The desulfurization rate of the total sulfur, pyrite sulfur removal rate, and deashing rate have all been significantly improved, increasing by 13.49% to 49.18%, 17.01% to 62.03%, and 33.40% to 33.63%, respectively. Therefore, a combined microwave magnetic separation–flotation desulfurization and deashing experiment should be selected.

## 3. Discussion

### 3.1. Phase Analysis

**Figure 16:**
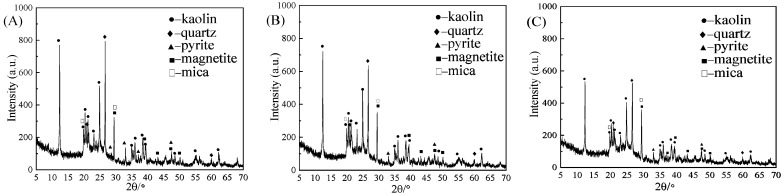
XRD curves of magnetic separation, microwave magnetic separation, and microwave magnetic separation–flotation ((**A**): magnetic separation, (**B**): microwave magnetic separation, and (**C**): microwave magnetic separation–flotation).

Figure 16 shows the XRD characterization results before and after microwave irradiation. (A) is the magnetic separation image, (B) is the microwave magnetic separation image, and (C) is the microwave magnetic separation–flotation image. The position and intensity of the diffraction peaks of (A), (B), and (C) are roughly the same. However, the intensity of the characteristic peaks decreases at the 2θ angles 33.55° and 47.55° and disappears at 27.85° and 37.12° for (A) and (B). It shows that pyrite is greatly reduced and transformed into magnetite, which enhances its magnetic properties and improves the magnetic separation effect. Therefore, increasing microwave irradiation before magnetic separation can improve desulfurization efficiency. From the analysis of (B) and (C), there is no disappearance of the characteristic peaks, but the intensity changes. It is indicated that the increase in the flotation experiment process will affect the grain change, so the desulfurization effect of microwave magnetic separation–flotation is the best.

### 3.2. Infrared Spectroscopic Analysis

**Figure 17:**
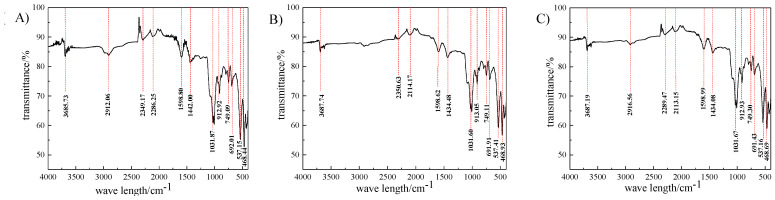
FTIR curves of magnetic separation, microwave magnetic separation, and microwave magnetic separation–flotation ((**A**): magnetic separation, (**B**): microwave magnetic separation, and (**C**): microwave magnetic separation–flotation).

Figure 17 shows the Fourier transform infrared spectra before and after microwave irradiation. (A) shows the magnetic separation image, (B) shows the microwave magnetic separation image, and (C) shows the microwave magnetic separation–flotation image. At 3687 cm^−1^, there are many impurity peaks in the vibration peak position, which is due to the variety of compounds in the coal sample. At 1434 cm^−1^, the peak position is the C–S bond in the –CH_2_–SH structure, while at 1031 cm^−1^, the peak position is the stretching vibration of the S=O bond in the sulfoxide. The vibration of the -S and -SH bonds is caused near 537 cm^−1^ and 468 cm^−1^, respectively. From the three curves (A), (B), and (C), it can be seen that the positions of the vibration peaks are the same, indicating that the main components of the coal samples from the magnetic separation, microwave magnetic separation, and microwave magnetic separation–flotation are basically the same, indicating that the microwave has the advantage of desulfurization and quality preservation. After microwave magnetic separation, the absorption peak intensities of the coal samples at 3687 cm^−1^, 1434 cm^−1^, 1031 cm^−1^, 537 cm^−1^, and 468 cm^−1^ were weakened compared to direct magnetic separation, corresponding to the groups that caused the changes in the peak positions. It was found that microwave magnetic separation can remove more pyrite and very little iron filings and inorganic sulfur from coal. After microwave magnetic separation–flotation, on the basis of removing inorganic sulfur in coal, it also has a certain response to the sulfur-containing groups in organic sulfur.

**Figure 18:**
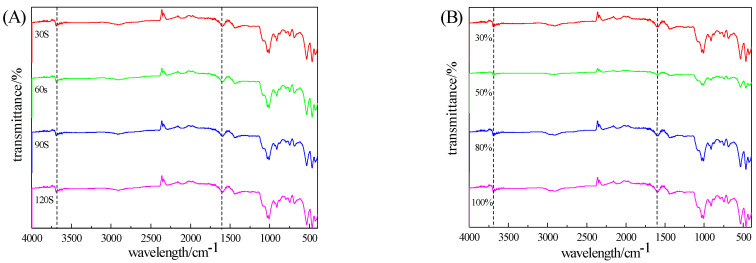
FTIR spectra of different irradiation times and different powers ((**A**): irradiation time; (**B**): irradiation power).

The motion laws of positive and negative charges in an electric field are different. The direction of the electric field force acting on positive charges is opposite to the direction of the electric field lines, while the direction of the electric field force acting on negative charges is the same as the direction of the electric field lines. When positive and negative charges come into contact, they may transfer from negative charges to positive charges, and the charge quotients will transform and exchange with each other, showing periodic changes. The electric charge vibrates and rubs back and forth in the circuit, and the thermal effect generated by this vibration and friction process will affect the structural changes in oxygen-containing functional groups in coal [24]. The Fourier transform infrared spectra with different irradiation times and powers are shown in Figure 18. (A) is the irradiation time image, and (B) is the irradiation power image.

It can be seen from Figure 18A,B that the characteristic peaks of the infrared spectra of the samples with different irradiation times and powers are basically the same, and the characteristic peaks of the main oxygen-containing functional groups are around 3686 cm^−1^ and 1698 cm^−1^. The characteristic peak near 3686 cm^−1^ is caused by the vibration of hydrogen-bonded –OH and –OH in free water. The characteristic peak near 1598 cm^−1^ is caused by the vibration of —C=O. After microwave irradiation, functional groups undergo mutual conversion, and the functional groups on the coal surface change. –OH and –COOH are the main hydrophilic functional groups in coal. With the increase in irradiation time and power, the characteristic peak of the oxygen-containing functional groups weakens, and their hydrophilicity decreases. The heating function of the microwave causes the water in the pores of the coal sample to evaporate, and the surface hydration effect weakens, thereby improving the effect of flotation desulfurization and deashing. At the same time, the characteristic of microwave selective heating disrupts the structure of coal and impurity minerals, creating cracks at the junction, which to some extent leads to an increase in the surface area of coal and enhanced hydrophobicity. So, after microwave irradiation, the magnetic separation effect of raw coal is enhanced, and it also has a positive promoting effect on flotation desulfurization.

### 3.3. Photoelectron Spectroscopy Analysis

**Figure 19:**
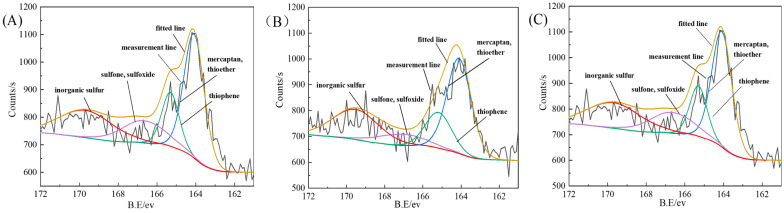
XPS curves of magnetic separation, microwave magnetic separation, and microwave magnetic separation–flotation ((**A**): magnetic separation, (**B**): microwave magnetic separation, and (**C**): microwave magnetic separation–flotation).

The existing forms of sulfur in coal are relatively complex, and the positions of sulfur peaks of the same type may vary due to the atomic types and structures, usually shifting within a relatively fixed range [25]. The peak positions of each sulfur form are thiol and thioether sulfur 162 ev~164 ev, thiophene sulfur 164 ev~165 ev, sulfone and sulfoxide sulfur 165 ev~168 ev, and inorganic sulfur 168 ev~171 ev. The X-ray photoelectron spectra before and after microwave irradiation are shown in Figure 19; (A) is the magnetic separation image, (B) is the microwave magnetic separation image, and (C) is the microwave magnetic separation–flotation image. The sulfur content of each type is shown in Table 3.

After fitting the fine spectrum of sulfur, the distribution of the sulfur content of the organic sulfur in raw coal is mercaptan, thioether > thiophene > sulfone, and sulfoxide. After microwave irradiation, some inorganic sulfur can be removed. After microwave magnetic separation–flotation, the sulfur content of various types in the clean coal changes, with a decrease in mercaptan, sulfide, thiophene, and inorganic sulfur and an increase in sulfone and sulfoxide sulfur. The main inorganic sulfur is pyrite sulfur with enhanced magnetism, which is removed by magnetic separation. However, mercaptan, sulfide, and thiophene sulfur in the organic sulfur component are oxidized to sulfone and sulfoxide, resulting in an increase in content.

## 4. Materials and Methods

### 4.1. Materials

(1) Reagent: Calcium oxide (CaO), analytical pure, Shenyang Quanrui Reagent Co., Ltd., Shenyang, China; kerosene (N/A), industrial grade, Shenyang Quanrui Reagent Co., Ltd., Shenyang, China; and pine alcohol oil (C_10_H_18_O), industrial grade, Shenyang Quanrui Reagent Co., Ltd., Shenyang, China. The experimental coal used is Shanxi coal.
(2) Instrument: Infrared sulfur analyzer, DNS-550 type, Beijing Dinai Instrument Co. Ltd., Beijing China; X-ray diffractometer, D8 ADVACE type, Brooke Instruments GmbH, Karlsruhe, Germany; scanning electron microscope, JSM-7610F type, JEOL Corporation, Tokyo, Japan; X-ray photoelectron spectrometer, Thermo Fisher ESCALAB XI+ type, Thermo Fisher Technology Co. Ltd., Waltham, MA, USA; wet intensity magnetic separator, XCSQ-50 × 70 type, Jiangxi Longzhong Machinery Equipment Co., Jiangxi, China; and microwave reactor, WBFY-201 type, Gongyi Yuhua Instrument Co. Ltd., Henan, China.

### 4.2. Analysis of Raw Coal Quality

The industrial analysis, elemental analysis, and form sulfur analysis of the raw coal are shown in Table 4 and Table 5.

**Figure 20:**
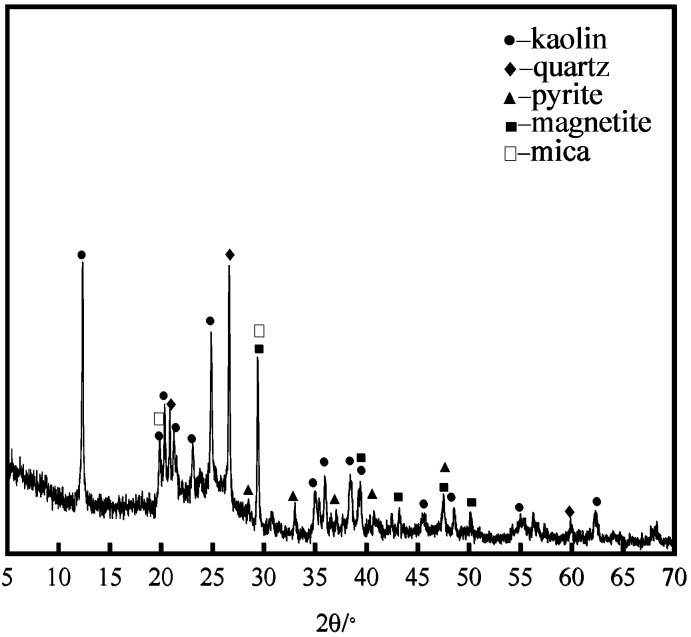
XRD plot of raw coal.

**Figure 21:**
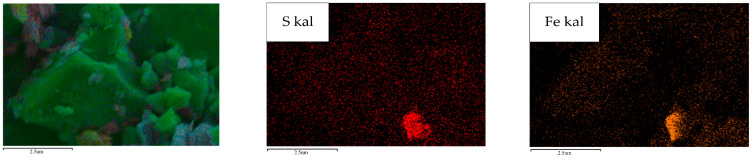
SEM-EDS energy spectrum of raw coal.

The phase analysis is shown in Figure 20 and the SEM-EDS energy spectrum is shown in Figure 21. Based on the phase analysis in Figure 20, it can be inferred that the distribution of the S and Fe elements in Figure 21 is pyrite FeS_2_ and troilite FeS.

### 4.3. Experiment Methods

(1) The coal sample is dried under natural conditions for 1 day, and the dried coal sample is broken to 3~0.5 mm. The sample was divided by the heap-cone quartering method. A total of 100 g per serving was bagged and sealed.
(2) The microwave magnetic separation experiments were carried out under the conditions of a grinding time of 0.5~12 min, microwave irradiation time of 30~120 s, microwave power of 30~100% of the rated power 800 W, magnetic field intensity of 2.25~2.35 T, and plate seam width of 1~4 mm.
(3) The refined coal obtained after microwave magnetic separation was poured into the flotation tank, the pulp concentration was adjusted to 50 g/L, and the stirring interval was 2 min. A foaming agent of 60–150 g/t, a collector of 1275–2550 g/t, and an inhibitor of 500–2000 g/t were added in sequence. After stirring for 1 min, the scraper of the flotation machine was opened, and the end time was when the clean coal was scraped completely.

This paper mainly studies the desulfurization and deashing effect of coal. Therefore, the desulfurization rate, deashing rate, and sulfur desulphurization rate of pyrite are taken as the evaluation indexes. According to Formulas (1)–(3), the desulfurization rate, the deashing rate, and the sulfur desulphurization rate of pyrite were calculated, respectively.

(1) $$S=\frac{100S_{y}-\gamma_{j}S_{j}}{S_{y}}$$

(2) $$A=\frac{100A_{y}-\gamma_{j}A_{j}}{A_{y}}$$

(3) $$w_{p}=\frac{(S_{W}-S_{y}\delta_{i})\gamma_{w}}{S_{y}\delta_{j}}\times 100\%$$

In the formula: *A_j_*—the clean coal ash content, %; *A_y_*—the ash content of raw coal, %; *S_j_*—the clean coal sulfur content, %; *S_w_*—the tail coal sulfur content, %; *S_y_*—the sulfur content of raw coal, %; *γ_j_*—the clean coal yield, %; *γ_w_*—the tail coal yield, %; *δ_i_*—the mass fraction of the non-pyrite sulfur in the total sulfur, %; *δ_j_*—the mass fraction of the pyrite sulfur in the total sulfur, %.

## 5. Conclusions

(1) Under the conditions of a microwave irradiation time of 60 s, a microwave power of 80% of the rated power (800 W), a grinding time of 8 min, a magnetic field strength of 2.32 T, a plate gap width of 1 mm, a slurry concentration of 50 g/L, a foaming agent dosage of 90 g/t, a collector dosage of 2125 g/t, and an inhibitor dosage of 1500 g/t, the desulfurization rate reached 76.51%, the pyrite sulfur removal rate was 96.50%, and the deashing rate was 61.91%.
(2) Microwave irradiation can enhance the magnetism of sulfur iron minerals in raw coal, improve magnetic separation efficiency, and to some extent improve the number of oxygen-containing functional groups and the hydrophobicity of minerals in coal. By combining microwave and magnetic separation and flotation, the comprehensive characteristics of clean coal are improved.
(3) A new process route for coal desulfurization has been provided through the experimental study of combined microwave magnetic separation–flotation coal desulfurization. And this method is effective and capable of removing the vast majority of inorganic sulfur. The limitation is that it cannot effectively remove organic sulfur. The follow-up direction can be to study chemical desulfurization methods and find a suitable method for the industrial large-scale removal of organic sulfur. This would allow for truly contributing to the green mountains and clear waters.

## Figures and Tables

**Table 1:** Analysis on total sulfur desulfurization rate, pyrite sulfur removal rate, and deashing rate of magnetic separation and microwave magnetic separation.

Experimental Method	Total Sulfur Desulfurization Rate	Sulfur Desulfurization Rate of Pyrite	Deashing Rate
magnetic separation	27.33%	34.47%	28.28%
microwave magnetic separation	63.02%	79.49%	28.51%

**Table 2:** Analysis on desulphurization rate, pyrite sulfur removal rate, and deashing rate of magnetic separation, microwave magnetic separation, and microwave magnetic separation–flotation.

Experimental Method	Total Sulfur Desulfurization Rate	Sulfur Desulfurization Rate of Pyrite	Deashing Rate
magnetic separation	27.33%	34.47%	28.28%
microwave magnetic separation	63.02%	79.49%	28.51%
microwave magnetic separation–flotation	76.51%	96.5%	61.91%

**Table 3:** Various forms of sulfur and their distribution in coal before and after experiment.

Experimental Method	Type	Binding Energy/ev	Peak Area Ratio/%
magnetic separation	mercaptan, thioether	164.10	46.14
	thiophene	165.38	17.87
	sulfone, sulfoxide	167.01	8.51
	inorganic sulfur	169.67	27.48
microwave magnetic separation	mercaptan, thioether	164.11	45.13
	thiophene	165.18	18.00
	sulfone, sulfoxide	167.10	9.82
	inorganic sulfur	169.54	27.05
microwave magnetic separation–flotation	mercaptan, thioether	164.09	42.51
	thiophene	165.30	15.83
	sulfone, sulfoxide	166.67	16.54
	inorganic sulfur	169.69	25.13

**Table 4:** Industrial analysis and elemental analysis of raw coal.

Industrial Analysis/%				Elemental Analysis/%				
M_ad_/%	A_ad_/%	V_daf_/%	FC_ad_/%	C_ad_/%	H_ad_/%	O_ad_/%	N_ad_/%	S_t,ad_/%
1.69	34.87	38.76	38.85	58.74	17.79	20.12	0.64	2.51

M_ad_ (Moisture): Moisture refers to the amount of water contained in a substance. A_ad_ (Ash): It refers to the inorganic substances remaining after coal combustion. V_daf_ (Volatile matter): It refers to the gas produced by the decomposition of organic matter contained in coal. FC_ad_ (Fixed carbon): Fixed carbon refers to the residue after subtracting volatiles from the total carbon. The total sulfur content on an air-dry basis is 2.51%, sulfate sulfur is 0.08%, sulfur content of pyrite is 1.99%, organic sulfur is 0.44%, and inorganic sulfur is 2.07%.

**Table 5:** Raw coal morphology sulfur analysis.

S_t,ad_/%	S_s,ad_/%	S_p,ad_/%	S_o,ad_/%
2.51	0.08	1.99	0.44

## Data Availability

The data presented in this study are available on request from the corresponding author.
